# A novel injectable BRET-based *in vivo* imaging probe for detecting the activity of hypoxia-inducible factor regulated by the ubiquitin-proteasome system

**DOI:** 10.1038/srep34311

**Published:** 2016-10-04

**Authors:** Takahiro Kuchimaru, Tomoya Suka, Keisuke Hirota, Tetsuya Kadonosono, Shinae Kizaka-Kondoh

**Affiliations:** 1 School of Life Science and Technology, Tokyo Institute of Technology, 4259-B60, Nagatsuta-cho, Midori-ku, Yokohama, 226-8501, Japan

## Abstract

The ubiquitin-proteasome system (UPS) is a selective protein degradation system that plays a critical role in many essential biological processes by regulating the existence of various cellular proteins. The target proteins of UPS are recognized and tagged with polyubiquitin chains by E3 ubiquitin ligases, which have high substrate-specific activities. Here we present a novel injectable imaging probe POL-N that can detect the UPS-regulated hypoxia-inducible factor (HIF) activity *in vivo*. Because the luciferase is fused to the E3 ligase-recognition domain of the HIF-1α, POL-N is intact only in the HIFα-overexpressing cells, that is, HIF-active cells, generating signals via an intramolecular bioluminescence resonance energy transfer (BRET) between luciferase and a near-infrared (NIR) fluorescent dye at the C-terminal end of the probe. Off-target signals of the NIR-BRET were so low that we could achieve highly sensitive and fast detection of intratumoral HIF-activity. Notably, we successfully detected hypoxic liver metastasis, which is extremely difficult to detect by injectable imaging probes due to strong off-target signals, as early as 1 h after systemic injection of POL-N. Our probe design can be widely adapted to UPS-target proteins and may contribute to the exploration of their roles in animal disease models.

To maintain cellular homeostasis, proteins must be recognized and degraded when their function is no longer appropriate. In UPS, one of the most important systems for selective protein degradation, the target proteins are tagged with polyubiquitin chains and are hydrolyzed into small peptides by the 26S proteasome in an energy-dependent manner^1^. Selective degradation via UPS is a complex process that involves several steps and is mediated by a series of enzymes including ubiquitin-activating enzyme (E1), ubiquitin-conjugating enzyme (E2), and ubiquitin ligase (E3)^1^.

Substrate recognition for ubiquitination is highly specific and is mediated by the physical interaction between E3 and E3-binding motifs in the substrate protein^1^. Therefore, fusing the motifs to certain proteins can endow the fused proteins with the same UPS regulation as the E3 substrate proteins. By most effectively using E3-binding motifs, several transgenic reporter systems have been constructed and successfully monitor cellular events regulated by UPS in real time. A striking example is the fluorescent ubiquitination-based cell-cycle indicator (Fucci) system for visualizing the cell-cycle status of living cells^2^ and transgenic mice^3^. Furthermore, reporter genes encoding a firefly luciferase have been fused to E3-binding motifs of the transcription factor NF-E2-related factor 2 (Nrf2) and hypoxia-inducible factor (HIF)-1α to provide spatiotemporal information about oxidative and hypoxic stress, respectively, in living mice^4^,^5^,^6^. In spite of the benefits of these reporter genes, a transgenic reporter system has limited applications; the signals from fluorescence proteins and luciferases are not long enough (λmax < 600 nm) to noninvasively detect targets in deep tissues of living animals; reporter genes have to be introduced to model animals, which is not always successful. Therefore, to further elucidate the functions of factors regulated by UPS *in vivo*, alternative injectable imaging probes are in high demand.

Noninvasive *in vivo* optical imaging has been widely used in biomedical research following the development of advanced imaging probes in NIR wavelength range where light signals are highly penetrable in biological tissues^7^,^8^. The goal of noninvasive *in vivo* optical imaging is to maximize the specific signals from deep tissues and minimize the background signals originating from off-target tissues. BRET is a promising technology that needs no excitation light and therefore minimizes tissue autofluorescence^9^,^10^,^11^,^12^, achieving a high target-to-background (T/B) ratio^13^.

Here we suggest an injectable NIR-BRET imaging probe design for the *in vivo* detection of the activity of factors regulated by UPS. As an archetypical example, we presented a probe POL-AF that enables highly specific (T/B ratio is approximately 10) and fast (<1 h after the probe injection) noninvasive detection of HIF activity, which is deeply dependent on HIF-1α protein availability, in deep tissues. The results presented here suggest that our injectable probe design is widely applicable for noninvasive detection of factors whose activity is regulated by UPS.

## Results

### Design of injectable BRET probe regulated by HIF-1α-specific UPS

**Figure 1:**
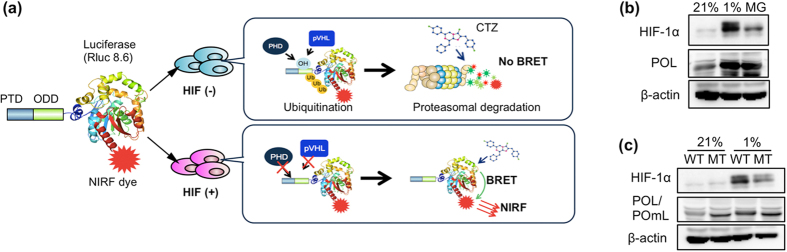
Design of injectable BRET probe regulated by HIF-1α-specific UPS. (**a**) A schematic diagram indicating the regulation of PTD-ODD-Luciferase-NIRF dye (POL-N) imaging probe in cells with (+) or without (−) HIF activity. CTZ, luciferase substrate coelenterazine. (**b**) Regulation of POL protein in cancer cells. SUIT-2 cells treated with POL were incubated for 30 min in normoxia (21% O_2_) or hypoxia (1% O_2_), or normoxia with proteasome-inhibitor MG132 (MG). Then protein levels of HIF-1α and POL were examined by western blotting with anti-HIF-1α and anti-Renilla luciferase antibodies, respectively. (**c**) SUIT-2 cells treated with POL (WT) or POmL (MT) are incubated for 30 min in normoxia (21% O_2_) or hypoxia (1% O_2_). Then POL or POmL was detected by western blotting. The ODD domain of POmL does not contain the proline residue, whose hydroxylation is required for VHL-recognition.

The oxygen-dependent degradation domain (ODD)_548–603_ of human HIF-1α contains an optimized VHL-recognition sequence^14^,^15^. The recombinant proteins containing ODD_548–603_ would be under the same UPS regulation as HIF-1α: They were stabilized in hypoxic cells and degraded in a VHL-dependent manner under normoxic conditions^14^,^15^. To achieve highly specific and fast detection of HIF activity in mice without using a transgenic reporter gene, we designed an injectable imaging probe PTD-ODD-luciferase-NIR fluorescent dye (POL-N), which generates NIR-BRET signals in a luciferase substrate-dependent manner, only when intact (Fig. 1a). In addition to ODD_548–603,_ POL has two other domains; a protein transduction domain (PTD)^16^ and red-shifted *Renilla* luciferase variant Rluc8.6-547^17^ (Supplementary Fig. S1). POL degradation was suppressed by the proteasome inhibitor MG132 and hypoxic treatment (Fig. 1b). Furthermore, a point mutation in the ODD, which corresponds to the P564G substitution mutation of human HIF-1α (Supplementary Fig. S1) and abolishes the ODD-regulation^18^, suppressed the degradation of POL in cancer cells under normoxic conditions (Fig. 1c). These results confirm that the stability of PTD-ODD fusion proteins was regulated by the UPS system through the ODD domain.

### Construction of NIR-BRET probe for specific detection of HIF activity

**Figure 2:**
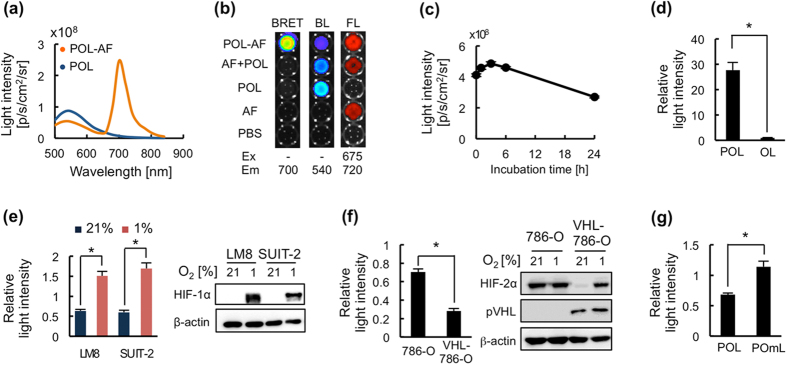
Construction of injectable BRET probe for specific detection of HIF activity. (**a**) Light spectrum of POL bioluminescence and POL-AlexaFluor680 (POL-AF) BRET signals. (**b**) BRET generation by POL labeled with a NIRF dye AF680 (AF). Samples containing POL conjugated with AF (POL-AF), mixture of POL and AF (AF + POL), POL, or AF were observed in a 96-well plate for their light generation with indicated excitation (Ex) and emission (Em) filters. BL, bioluminescence; FL, fluorescence. (**c**) POL-AF stability in serum at 37 °C. POL-AF was incubated with 50% fetal calf serum (FCS) at 37 °C and BRET signal intensity was measured at indicated time by using IVIS-Spectrum with a filter of 700 nm. (**d**) Efficient delivery of POL-AF into cells through PTD function. LM8 cells were treated with POL-AF (POL) or OL-AF (OL), which lacks PTD sequences, for 30 min and then the harvested cells were examined their generation of BRET signals. The signals were calibrated based on the data shown in Fig. S3. The calibrated signal intensity from POL-treated cells was normalized by that of OL-treated cells. (**e**) Specific generation of BRET signals by POL-AF in hypoxic cells. Cancer cells were treated with POL-AF for 30 min under normoxic (21% O_2_) or hypoxic (1% O_2_) conditions and then BRET signals in the harvested cells were measured. (**f**) VHL-dependent regulation of BRET in 786-O cells. 786-O and VHL-reconstituted 786-O (VHL-786-O) cells are used in the assay. Cancer cells were treated with POL-AF for 30 min under normoxic (21% O_2_) conditions and then BRET signals in the cells were measured. (**g**) Oxygen-independent generation of BRET signal from POL with mutated ODD domain. LM8 cells treated with POL-AF or POmL-AF were incubated for 30 min under normoxic conditions and then BRET signals were measured. Relative BRET intensity in (**e–g**) was calculated by dividing signal intensities of the 1 h-cultured cells, which were incubated with fresh medium for 1 h followed by 30 min incubation with probes, by their corresponding signal intensity of the uncultured cells, which were trypsinized immediately after 30 min incubation with the probes (see Methods for details). n = 3, *p < 0.05. Error bars indicate s.e.m.

The bioluminescence of Rluc8.6-547 in POL has a peak at 547 nm^17^. For efficient generation of the BRET signal in the NIR wavelength range, POL was labeled with a suitable NIR fluorescent dye, AlexaFluor 680 (AF), which is excited with a peak around 684 nm and fluoresces with a peak around 700 nm, making the large overlap between the donor (luciferase Rluc8.6-547) bioluminescence and the acceptor (AF) absorption spectra (Supplementary Fig. S2a). A maleimide-AF was conjugated with POL at the C-terminal cysteine residue (C-Cys), which was inserted because its internal Cys residues are not exposed on the surface (Supplementary Fig. S2b) and POL was not labeled without C-Cys (Supplementary Fig. S2c). AF-labeled POL, named as POL-AF, efficiently generated BRET signals with a peak around 700 nm in the presence of coelenterazine (Fig. 2a) and signal intensity was POL-AF concentration-dependent (Supplementary Fig. S3a). The BRET signal from POL-AF was intra-molecularly generated (Fig. 2b) and completely extinguished upon treatment with the proteolytic enzyme, trypsin (Supplementary Fig. S4a), although AF fluorescent signals from trypsin-treated and untreated POL-AF were not changed (Supplementary Fig. S4b), confirming that the signal was generated by BRET between POL and AF. The BRET signal (λmax = 702 nm) generated from POL-AF efficiently penetrated biological tissues than the original bioluminescence (λmax = 547 nm) of POL (Supplementary Fig. S5), suggesting the superiority of NIR-BRET signal in *in vivo* imaging. POL-AF was stable and could generate a BRET signal after 24 h incubation at 37 °C in serum (Fig. 2c), indicating that the probe may be applicable for the sequential analysis of target factors *in vivo* as well as in cultured cells.

POL-AF was evaluated using cultured mouse osteosarcoma LM8/HRE-Fluc and human pancreatic cancer SUIT-2/HRE-Fluc cells, which stably carry HIF-dependent firefly luciferase reporter gene^19^. PTD was confirmed to be indispensable for effective delivering of POL-AF probes into cells by comparing OL-AF which lacks PTD (Fig. 2d and Supplementary Fig. S3b). PTD function is also important in efficient delivery of PTD-fusion protein to tumors through Neuropilin-1-dependent extravasation^20^. The correlation between the BRET signal intensity and HIF-1α protein expression was examined in these cells treated with POL-AF under normoxic or hypoxic conditions. The BRET signal intensity quickly decreased in normoxic cells, in which HIF-1α was not detected, and increased in hypoxic cells, in which HIF-1α accumulated (Fig. 2e). HIF-dependent bioluminescence intensities in LM8/HRE-Fluc and SUIT-2/HRE-Fluc cells correlated well with an oxygen-dependent regulation of BRET signal intensity (Fig. 2e and Supplementary Fig. S6), indicating that the POL-AF probe can detect HIF-1-active cells. Because both HIF-1α and HIF-2α are regulated by UPS through VHL^21^, although the ODD motif of POL is from HIF-1α ODD domain, POL-AF may be able to detect HIF-2-active cells if these cells have the basically same PHD hydroxylation and VHL recognition. To address this possibility, we examined the difference in BRET signal generation between 786-O, which is a VHL-null cell line constitutively expressing HIF-2α but not HIF-1α, and VHL-786-O, which expresses wild-type VHL and exhibited oxygen-dependent expression of HIF-2α (Fig. 2f). Higher BRET signals were observed in 786-O cells compared with those in VHL-786-O cells under normoxic conditions (Fig. 2f), indicating that the POL-AF probe can also detect HIF-2-active cells. Furthermore, POmL-AF that has the mutated ODD^18^ did not reduce the BRET signal intensity in normoxic cells (Fig. 2g), confirming that POL-AF was regulated by the VHL-mediated UPS (Fig. 1b,c). These results demonstrated that POL-AF was regulated by the HIFα-E3-mediated UPS and that the BRET signals generated from POL-AF strongly correlated with HIF-1 and HIF-2 activities.

### Fast and specific detection of HIF activity *in vivo*

**Figure 3:**
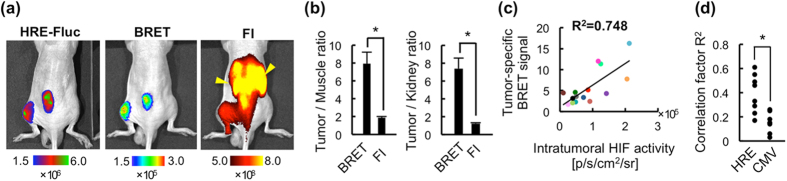
*In vivo* imaging of subcutaneous tumors using POL-AF. (**a**) Representative images of HIF activity (HRE-Fluc), BRET and fluorescence imaging (FI). BRET and FI images were obtained 1 h after i.v. injection of POL-AF and the HRE-Fluc image was obtained 3 h after BRET imaging. Yellow arrowheads in FI image indicate fluorescence signals from the kidneys. (**b**) Quantitative analysis of tumor-specific BRET signals from POL-AF. Tumor/Muscle and Tumor/Kidney ratio of BRET and FI signals 1 h after POL-AF injection are shown. n = 6, *p < 0.05. Error bars indicate s.e.m. (**c**) Correlations between intratumoral HIF-activity (HRE-Fluc bioluminescence) and tumor-specific BRET signals (T/M ratio) 1 h after injection of POL-AF. Each color circle indicates one tumor. n = 17. The results of the same subcutaneous tumors were indicated by the same colored circles. (**d**) Correlation of spatial intensity distribution between POL-AF BRET and HRE-Fluc or CMV-Fluc bioluminescent images. n = 8. *p < 0.05.

The correlation between BRET signals and intratumoral HIF activity was examined 15 min and 1 h after the intravenous (i.v.) administration of POL-AF, when the detection of targets is almost impossible with conventional fluorescence imaging (FI) using a NIR-fluorescent probe, to LM8/HRE-Fluc subcutaneous tumor-bearing mice (Fig. 3a). Strong signal was observed in the whole body with both BRET and FI 15 min after POL-AF injection (Supplementary Fig. S7). Although it was impossible to detect HIF-specific signal by FI due to strong off-target signals, BRET signals from the same mice were highly correlated with the HIF-dependent bioluminescent signals due to quick clearance of off-target signals 1 h after POL-AF injection (Fig. 3a and Supplementary Fig. S7). Notably, BRET imaging dramatically reduced off-target signals from the circulation, excretory organs including the kidney and liver compared with those in FI, allowing for the detection of HIF activity in tumors by increasing tumor/muscle and tumor/kidney ratios in BRET imaging compared with those in FI (Fig. 3a,b and Supplementary Fig. S8a). *Ex vivo* imaging further confirmed that fluorescent signals over 10-fold higher were detected in the kidney and liver compared with those in the tumors 1 h after i.v. injection of POL-AF (Supplementary Fig. S8b). Furthermore, BRET signals quickly became undetectable in circulating blood (Supplementary Fig. S8c). These results revealed that most of the signals detected by FI in the excretory organs and blood circulation 1 h after the probe administration were not from the intact probe but were from dyes released from POL probes degraded in HIF-inactive cells. This demonstrates that this BRET-based imaging probe design is ideal one for detecting the activity of a factor regulated by UPS because the BRET signal disappears by probe degradation, which would occur in parallel with the UPS-target protein degradation.

### Specificity of BRET to intratumoral HIF activity

**Figure 4:**
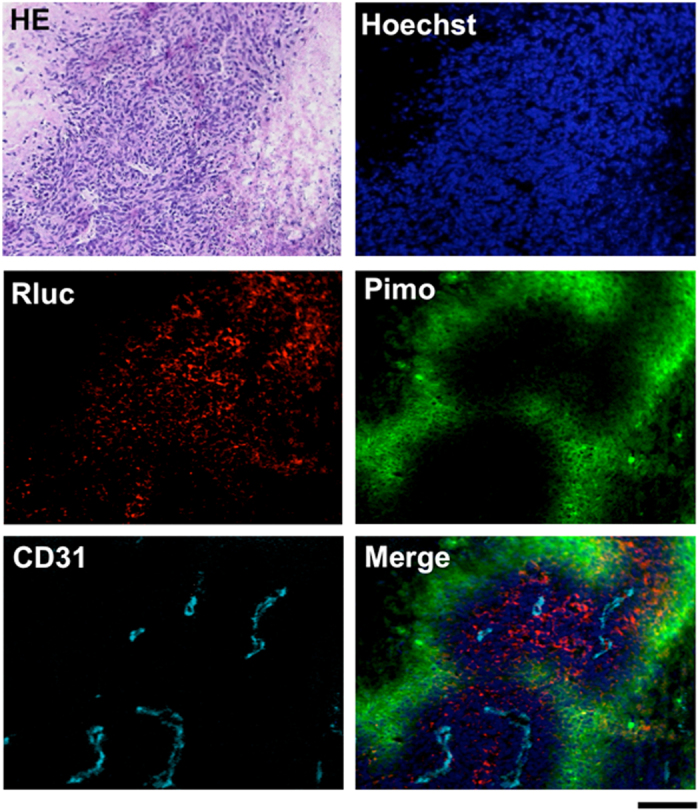
Immunohistochemical analysis of subcutaneous tumors injected with POL-AF. The tumor was removed 1 h after i.v. injection of POL probe and then frozen sections were prepared and stained with Hoechst and antibodies against Renilla luciferase (Rluc), CD31, and pimonidazole (Pimo). Bar = 100 μm.

The BRET signal from tumors 1 h after POL-AF injection strongly correlated with intratumoral HIF activity (Fig. 3c), whereas the fluorescent signal from tumors poorly correlated (Supplementary Fig. S9), probably due to strong off-target signals. Correlations between the BRET signal and HIF activity were further evaluated by comparing the spatial intensity distribution of the BRET signals with one of the bioluminescence signals in tumors carrying the HRE-Fluc or CMV-Fluc reporter gene. Significantly higher correlations were observed between tumor-specific BRET and HIF-dependent bioluminescence signals from HRE-Fluc tumors when compared with those between the tumor-specific BRET and bioluminescence signals from CMV-Fluc tumors (Fig. 3d). In addition, immunohistochemical analysis of tumors 1 h after POL-AF administration confirmed that the probe was mainly detected close to the pimonidazole-positive hypoxic regions (Fig. 4 and Supplementary Fig. S10a) and overlapped with the HIF-active regions (Supplementary Fig. S10b). Consistent with the previous observation^18^, the probe lacking a nuclear localization signal (NLS) was localized in the cytoplasm, while HIF-1α, which has two NLS sequences^22^, was in the nucleus (Supplementary Fig. S10c). Overall, the results indicate that POL-AF imaging probe achieved highly specific and fast detection of intratumoral HIF activity *in vivo*.

### Application of HIF-specific BRET probe

**Figure 5:**
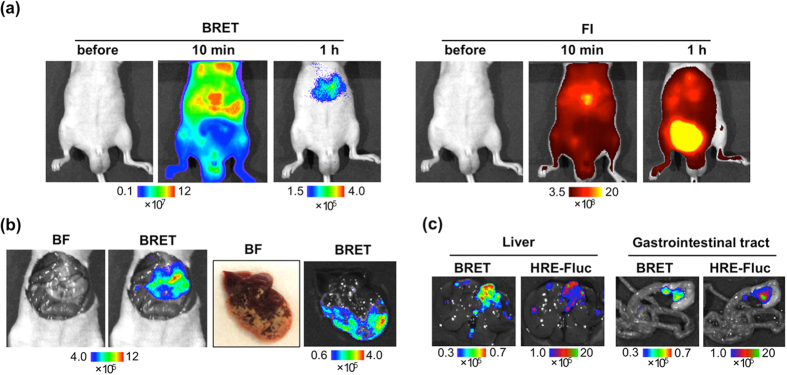
*In vivo* liver metastasis imaging by BRET. (**a**) Liver metastasis BRET and FI images 10 min and 1 h after POL-AF injection. Mice was injected Colon-26/HRE-Fluc into the spleen 5 days before *in vivo* imaging. (**b**) *Ex vivo* images of the mouse shown in (**a**). Bright field (BF) and BRET images of the liver are shown. (**c**) *Ex vivo* images of SUIT-2/HRE-Fluc peritoneal dissemination model 3 h after i.v. injection of POL-AF (1 nmol). A mouse was imaged 20 days after intraperitoneal injection of cancer cells.

Taking advantage of the specific and fast detection of intratumoral HIF activity using the BRET signal of POL-AF, we challenged the noninvasive detection of metastasis using POL-AF in liver metastasis and peritoneal dissemination models. In these models, extremely high off-target signals from the liver, gastrointestinal tract, and bladder hamper early and specific detection of metastasis by using a NIR-fluorescent probe and FI. The murine colorectal cancer cell Colon-26 stably carrying an HRE-Fluc reporter (Colon-26/HRE-Fluc) that expresses firefly luciferase (Fluc) in a HIF-dependent manner (Supplementary Fig. S11a) was injected into the spleen of nude mice to induce liver metastasis. Although strong off-target signals hampered fluorescence signals from the tumors using FI, the BRET signal from POL-AF allowed us to detect metastasis in the liver 1 h after the POL-AF injection (Fig. 5a). *Ex vivo* imaging confirmed that the BRET signals were specifically generated from the metastasis that contained HIF-active regions (Fig. 5b and Supplementary Fig. S11b). Poor blood flow in liver metastasis was confirmed by a significantly low perfusion of Hoechst dyes, implying that the metastatic region is hypoxic (Supplementary Fig. S11c,d). Furthermore, we successfully detected small metastases in the liver and gastrointestinal tract *ex vivo* by the BRET signal in a peritoneal dissemination model (Fig. 5c). BRET images correlated well with the HIF-dependent bioluminescence images.

## Discussion

In this study, we developed an injectable BRET-based imaging probe, which achieved highly specific and fast detection of intratumoral HIF activity in various cancer models. The data presented here demonstrate that the probe design that allows only intact probe to generate signals is suitable for analyzing *in vivo* activity of factors regulated by UPS.

In contrast to HIF-1β that is constantly expressed, expression of HIF-1α is regulated by many mechanisms at the transcriptional, translational, and post-translational levels^23^. Therefore, HIF-1 activity is mostly dependent on the availability of HIF-1α. The total accumulation of HIF-1α is a consequence of the balance between its expression and UPS-mediated degradation. In normal cells, the degradation level dominates over the expression level under normoxic conditions; HIF-1α is expressed but undetectable due to O_2_-dependent UPS-mediated degradation, and it accumulates under hypoxic conditions because HIF-1α modification by prolyl hydroxylase for E3 recognition does not occur^24^. In cancer cells, however, the expression level often dominates over the degradation level. HIF-1α can accumulate in normoxic cells through various mechanisms including mutations in tumor suppressor genes such as VHL^25^ and p53^26^, and aberrant Ras/Raf/MAPK and PI3K/AKT/mTOR signaling, which increase intracellular HIF-1α level, leading to an overload in UPS processing^27^. The results of Fig. 1b,c support that POL is regulated by the same UPS as HIF-1α and shares its fate with HIF-1α; that is, POL would accumulate in HIF-active cells and is degraded quickly in HIF-inactive cells (Fig. 1a).

Our study demonstrates that an intramolecular BRET signal generated between a single donor and single acceptor is enough for *in vivo* imaging. To date, intermolecular BRET generated between multiple luciferase donors and a large quantum dot acceptor has been used for molecular imaging. Although highly efficient BRET is expected to be generated among molecules and to show excellent performance in lymph node mapping^10^,^13^,^28^, it would be difficult to image intracellular events using these probes because of inefficient membrane transduction of nanoparticle-based probes.

A combination of BRET technology and E3-binding motifs dramatically reduced off-target signals from the whole body including the excretory organs, resulting in highly specific and fast detection of the target *in vivo* (Figs 3 and 5). Because many factors regulated by UPS play a critical role in several important aspects of cell biology, their aberrant activation or deactivation leads to the development of distinct human diseases including cancers. Our study suggests a general strategy to design highly specific injectable imaging probes for detecting such aberrant states of the factors *in vivo* as well as *in vitro*. Injectable imaging probes can be applied to any models and to any timing on demand without genetic manipulation and thus may be used to obtain information that was previously unobtainable by using genetically engineered reporter probes. Therefore, this study would open new avenues for molecular imaging.

## Methods

### Plasmid construction

Rluc8.6-547 cDNA was obtained by introducing 14 mutations^17^ into pRL-CMV (Promega, Madison, WI, USA) by site-directed mutagenesis. Furthermore, a cysteine residue was inserted at the C-terminal of Rluc8.6-547 to obtain Rluc8.6-547-Cys. The plasmid encoding PTD-ODD-Luciferase was constructed by substituting coding sequence of Halotag in the PTD-ODD-Halotag^18^ to Rluc8.6-547-Cys.

### Preparation of POL-AF probe

PTD-ODD-Luciferase (POL), ODD-Luciferase (OL) and PTD-ODDmutant-Luciferase (POmL) fusion proteins were expressed in BL21-CodonPlus cells (Stratagene, La Jolla, CA, USA) as GST-tagged fusion proteins. The GST-tagged proteins were prepared essentially as described previously^18^ and purified with a GST-column and digested with precision protease (GE Healthcare, Waukesha, WI, USA) to remove GST-tag from the fusion protein. The final products were equilibrated in Mg/Ca-free PBS (pH = 7.4). The fusion proteins (20 nmol/mL) were mixed with Alexa Fluor 680 C-2 maleimide (60 nmol/60 μL) (Invitrogen, Carlsbad, CA, USA) at 4 °C for 12 h. POL-AF probes were purified with a PD-10 gel filtration column (GE Healthcare) and an Amicon-10 centrifugation column (Millipore, Milford, MA, USA). The purified POL-AF was finally resolved in PBS (pH 7.4). Fluorescence-labeling characterizations were confirmed by SDS-PAGE fluorescence imaging and the labeling rate was calculated by colorimetric method with dye dilution series. The labeling rate was >0.9.

### Light spectrum measurement of POL-AF

Light spectrum of POL-AF was analyzed with IVIS-Spectrum (PerkinElmer, Norwalk, CT, USA). POL-AF and coelenterazine (final concentration of 5 nM and 12 μM, respectively) (Chisso, Tokyo, Japan) were reacted in 96-well plate and then light intensity was measured with 18 emission filters from 500 nm to 840 nm (20 nm bandwidth). To minimize influence of reaction kinetics of BRET, final spectrum was calculated by averaging with light intensity measured with same filter sets from 840 nm to 500 nm.

### Cell culture

The murine osteosarcoma cell line LM8 was gifted from Dr. Yoshikawa (Osaka University, Japan). Human pancreatic cancer cell line SUIT-2 was purchased from Japanese Cancer Research Resource Bank and, human renal cancer cell line 786-O and mouse colorectal cancer cell line Colon-26 were purchased from ATCC. HRE-Fluc reporter gene was transduced to these cell lines by calcium phosphate method and isolated from a colony after antibiotic selection as described previously^16^,^29^,^30^. VHL-expressing 786-O cell line was also isolated from a colony after transfection with a plasmid encoding VHL followed by antibiotic selection. The plasmid coding VHL was constructed by insertion of VHL sequence into pcDNA3.1 plasmid (Invitrogen). The cells were maintained at 37 °C in 5% FCS-DMEM (Nacalai Tesque, Kyoto, Japan) supplemented with penicillin (100 units/mL) and streptomycin (100 μg/mL).

### Cellular assay

The cells (1 × 10^5^ cells/well) were seeded into 6-well plates and preincubated in normoxia (21% O_2_) or hypoxia (1% O_2_) for 12 h. After the addition of POL imaging probes (final concentration of 100 pM), the cells were incubated for 30 min under the same conditions as preincubation. Then, the cells were immediately washed with PBS and trypsinized for 5 min at 37 °C (uncultured cells) or further incubated for 1 h with fresh medium under the same conditions as preincubation, followed by trypsinization (1 h cultured cells). Trypsinized cells were suspended in 50 μL PBS after centrifugal recovery. Then, 50 μL of 24 μM coelenterazine was added to the cell suspension in 96-well plate to measure BRET signal intensity by IVIS. Relative BRET signal intensity was calculated by normalizing the BRET signals of the 1 h-cultured cell samples with the one of the uncultured cell samples. For hypoxic samples, all procedures were performed in the *in vivo* Hypoxia Workstation (Ruskinn Technology, Leeds, UK). Image analysis was performed with Living Image 4.2.

### Westernblotting

The cells (2.0 × 10^5^ cells/well) were seeded in a six-well plate. The cells were pre-incubated under normoxic or hypoxic conditions for 16 h, or normoxic with MG132 (50 μM) (Wako, Tokyo, Japan) treatment for 4 h. Then, POL probe (final concentration of 100 nM) was added to the culture medium and the cells were incubated for 30 min under the same conditions as preincubation. The cells were then washed with fresh medium and further incubated for 1 h under the same conditions. Total cell lysates were electrophoresed on a 12.5% SDS-polyacryl-amide gel and transferred to a Hybond ECL membrane (GE Healthcare). The membrane was treated with primary antibodies such as monoclonal anti-HIF-1α antibody (R&D systems, Abingdon, UK), polyclonal anti-HIF-2α antibody (Novus Biologicals, Litteleton, CO, USA), polyclonal anti-Renilla luciferase antibody (Biocompare, South San Francisco, CA, USA), polyclonal anti-pVHL antibody (Cell Signaling Technology, Beverly, MA, USA), monoclonal anti-actin antibody (Sigma, St. Louis, MO, USA). The primary antibodies bound to their targets on the membrane were then probed using appropriate secondary antibodies conjugated with horseradish peroxidase (GE Healthcare) and chemiluminescent Chemi-Lumi One system (Nacalai Tesque).

### Tumor models

Male Balb/c nu/nu mice were purchased from Oriental Yeast Co., Ltd. All mice underwent experiments at 6–10 weeks of age. For subcutaneous tumors, LM8/HRE-Fluc cells suspended in PBS (1 × 10^6^ cells/20 μL) were mixed with an equal volume of Geltrex (Invitrogen) and injected into the back and hind limb of nude mice. For the liver metastasis model, Colon-26/HRE-Fluc (5 × 10^5^ cells/100 μL) was injected in the spleen of nude mice. For the peritoneal dissemination model, SUIT-2/HRE-Fluc (3 × 10^5^ cells/100 μL) was intraperitoneally injected into nude mice. Mice with subcutaneous tumors of 5–10 mm in diameter were used for experiments. As early liver metastasis model, we used mice at 5–7 days after injection of cancer cells. Meanwhile, for peritoneal dissemination model, we waited for 20 days after cancer cell injection. All animal experiments were performed with the approval of the Animal Experiment Committees of Tokyo Institute of Technology (authorization number 2010006) and conducted according to relevant national and international guidelines.

### *In vivo* BRET, fluorescence and bioluminescence imaging

One nmol of POL-AF in 100 μL of PBS was injected into tumor-bearing mice via tail vain. BRET images were acquired at the indicated times 5 min after injection of 10 μg coelenterazine via orbital vein. Sequentially, fluorescence reflectance images were acquired. Bioluminescence images with Fluc reporter systems were typically performed 3 h after obtaining BRET images. D-luciferin (50 mg/kg) was intraperitoneally injected to tumor bearing mice before acquisition of bioluminescence images. All of the BRET images and bioluminescence images were acquired with IVIS-Spectrum (PerkinElmer) using the same parameters: the excitation filter (block), emission filter (open), exposure time = 30 s for BRET imaging and 1 min for Fluc bioluminescence imaging, binning = small (4), field of view = 12.9 × 12.9 cm, f/stop = 1. For fluorescence reflectance images, the images were acquired with IVIS-Spectrum using the same parameters besides the excitation filter (675 nm), emission filter (720 nm), exposure time = 1 s. Image analysis was performed with Living Image 4.2. Tumor/Muscle ratio was calculated by using same size of ROI for a tumor and hind limb with no tumor. Similarly, Tumor/Kidney ratio was calculated by using same size of ROI for a tumor and the kidney. Correlation of spatial intensity distribution between BRET and Fluc reporter signals was analyzed with ImageJ 1.47 and Image CorrelationJ plugin^31^.

### *Ex vivo* BRET, fluorescence and bioluminescence imaging

Randomly selected mice were sacrificed immediately after the *in vivo* imaging and organs were harvested. BRET signal and fluorescence emissions from these organs were measured with the IVIS system using the same parameters for *in vivo* imaging. Sequentially, harvested organs were dipped into D-luciferin (10 mg/mL) to obtain HRE-Fluc bioluminescence images with 1 min exposure time. Image analysis was performed with Living Image 4.2.

### Histological analysis

For immunofluorescence staining of intratumoral hypoxic region, mice were intraperitoneally injected with 25 mg/kg Hypoxyprob-1 (Chemicon International Inc., Temucula, CA, USA) 1 h before dissection. Removal tumors were embedded in Tissue-Tek OCT compound (Sakura Finetechnical, Tokyo, Japan) in dry ice/methanol baths. Serial cryosections (10 μm thick) were prepared using a cryostat (Leica CM3050S, Leica Microsystems, Wetzlar, Germany). Serial cryosections were fixed in 4% paraformaldehyde for immunohistochemical analysis (Wako) and stained with hematoxylin-eosin for HE staining analysis. Then sections for immunohistochemical analysis were incubated with block solution (1% BSA PBS) for 30 min at room temperature. The primary antibodies, mouse anti-HIF-1α antibody (BD Transduction Laboratories, San Diego, CA, USA), rat anti-CD31 antibody (Dianova, Hamburg, Germany) and rabbit anti-Renilla luciferase antibody (Biocompare) were incubated. TRITC-conjugated goat anti-rabbit and Alexa Fluor 647-conjugated goat anti-rat (Invitrogen) secondary antibodies were used to fluorescently label the corresponding primary antibodies. For HIF-1α detection, Vector Mouse on Mouse kit was used (Vector Laboratories, Burlingame, CA, USA). FITC-conjugated Hypoxyprob-1 primary antibody supplied with the kit (Hypoxyprobe-1 Plus Kit, Chemicon International Inc.) was used to stained intratumoral hypoxic regions. Stained cross section was mounted with Fluoromount^TM^ (DBS, Pleasanton, CA) and Hoechest 33342 (Nacalai Tesque). All photos were taken using a BZ-8100 microscope (Keyence, Osaka, Japan).

### Statistical analysis

Data are presented as means ± standard error of the mean (s.e.m.) and were statistically analyzed with a two-side student’s t-test. P values of less than 0.05 were considered statistically significant. Pearson’s correlation coefficient was also calculated for evaluation of the correlation of data sets.

## Additional Information

**How to cite this article**: Kuchimaru, T. *et al*. A novel injectable BRET-based *in vivo* imaging probe for detecting the activity of hypoxia-inducible factor regulated by the ubiquitin-proteasome system. *Sci. Rep.* **6**, 34311; doi: 10.1038/srep34311 (2016).

## Supplementary Material

Supplementary Information
