# Supplementary material for: A novel injectable BRET-based in vivo imaging probe for detecting the activity of hypoxia-inducible factor regulated by the ubiquitin-proteasome system
Source: Sci Rep. 2016 Oct 4;6:34311. doi: 10.1038/srep34311 (PMC5048432; doi:10.1038/srep34311)
Supplement: Supplementary Information [file srep34311-s1.pdf]

Supplementary information

A novel injectable BRET-based *in vivo* imaging probe for detecting the activity of hypoxia-inducible factor regulated by the ubiquitin-proteasome system

Takahiro Kuchimaru, Tomoya Suka, Keisuke Hirota, Tetsuya Kadonosono, Shinae Kizaka-Kondoh

**Supplementary item**	**Title**
Figure S1	Amino acid sequence of PTD-ODD-Luciferase.
Figure S2	Construction of BRET imaging probes.
Figure S3	Correlations between probe concentration and intensities of light signals.
Figure S4	Abrogation of BRET in POL-AF by trypsin treatment.
Figure S5	Analysis of tissue penetration efficiency of BRET signal.
Figure S6	Regulation of transcriptional activity of HIF under normoxic or hypoxic conditions.
Figure S7	Time-course imaging of subcutaneous tumors of LM8.
Figure S8	Analysis of off-target signals for *in vivo* imaging of POL-AF.
Figure S9	Correlation between intratumoral HIF-activity and tumor-specific fluorescence signal of POL-AF.
Figure S10	Immunohistochemical analysis of subcutaneous tumors.
Figure S11	HIF-activity in liver metastasis of Colon-26/HRE-Fluc.

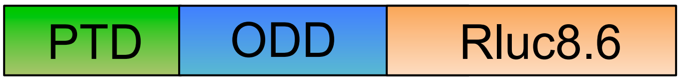

PTD → ODD →

GPLGSPNSKKKKKKKKKETWWETWWTEWRSGGKNPFST

QDTDLDLEMLAPYIPMDDDFQLRSFDQLSPLESSSASPES

Luciferase (Rluc8.6-547) →

ASPQSTVTVFQQVMASKVYDPEQRKRMITGPQWWARCK

QMNVLDSFINYYDSEKHAENAVIFLHGNATSSYLWRHVVP

HIEPVARCIIPDLIGMGKSGKSGNGSYRLLDHYKYLTAWFE

LLNLPKKIIFVGHDWGSALAFHYAYEHQDRIKAIVHMESVV

DVIESWAGWPDIEEEVALIKSEEGEKMVLENNFFVETLLPS

KIMRKLEPEEFAAYLEPFKEKGEVRRPTLSWPREIPLVKGG

KPDVVQIVRNYNAYLRASDDLPKLFIESDPGFWSNAIVEGA

KKFPNTEFVKVKGLHFLQEDAPDEMGKYIKSFVERVLKNE

Q

Supplementary Figure S1. Amino acid sequence of PTD-ODD-Luciferase (formula weight: 46.4 kDa). Amino acids of PTD, ODD and Luciferase are indicated with green, blue and orange color, respectively. A proline residue (P) indicated with red color was substituted to glycine in POmL protein.

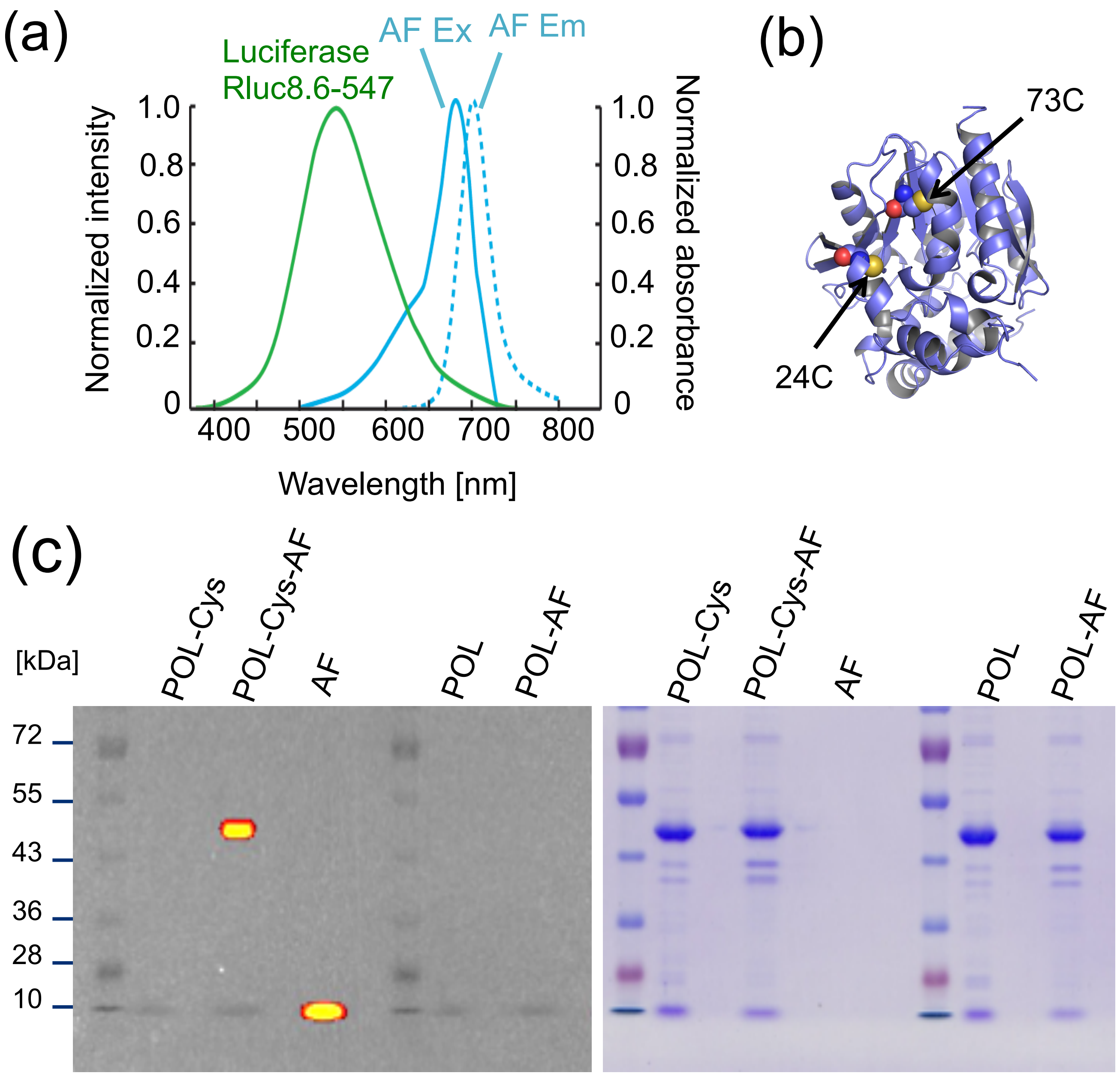

Supplementary Figure S2. Construction of BRET imaging probes. (**a**) The spectral overlap between the donor bioluminescence spectrum (luciferase Rluc8.6-547) and the acceptor AlexaFluor 680 (AF) absorption spectrum (AF Ex). Dashed line indicates AlexaFluor 680 emission spectrum (AF Em). (**b**) Three-dimensional modeling of Renilla luciferase 8 (Rluc8). The internal cysteine residues of Rluc8 are not exposed on the surface. Yellow spheres indicate sulfur atoms of cysteine residue. (**c**) Fluorescence labeling of probes. POL and POL with a cysteine at the C-terminal end (POL-Cys) were labeled with AF and the resultant probes (POL-AF and POL-Cys-AF) were evaluated by SDS-polyacrylamide gel electrophoresis. The probes in the gel were stained with Coomassie Brilliant Blue (right panel) and their fluorescence was detected by IVIS-Spectrum (left panel).

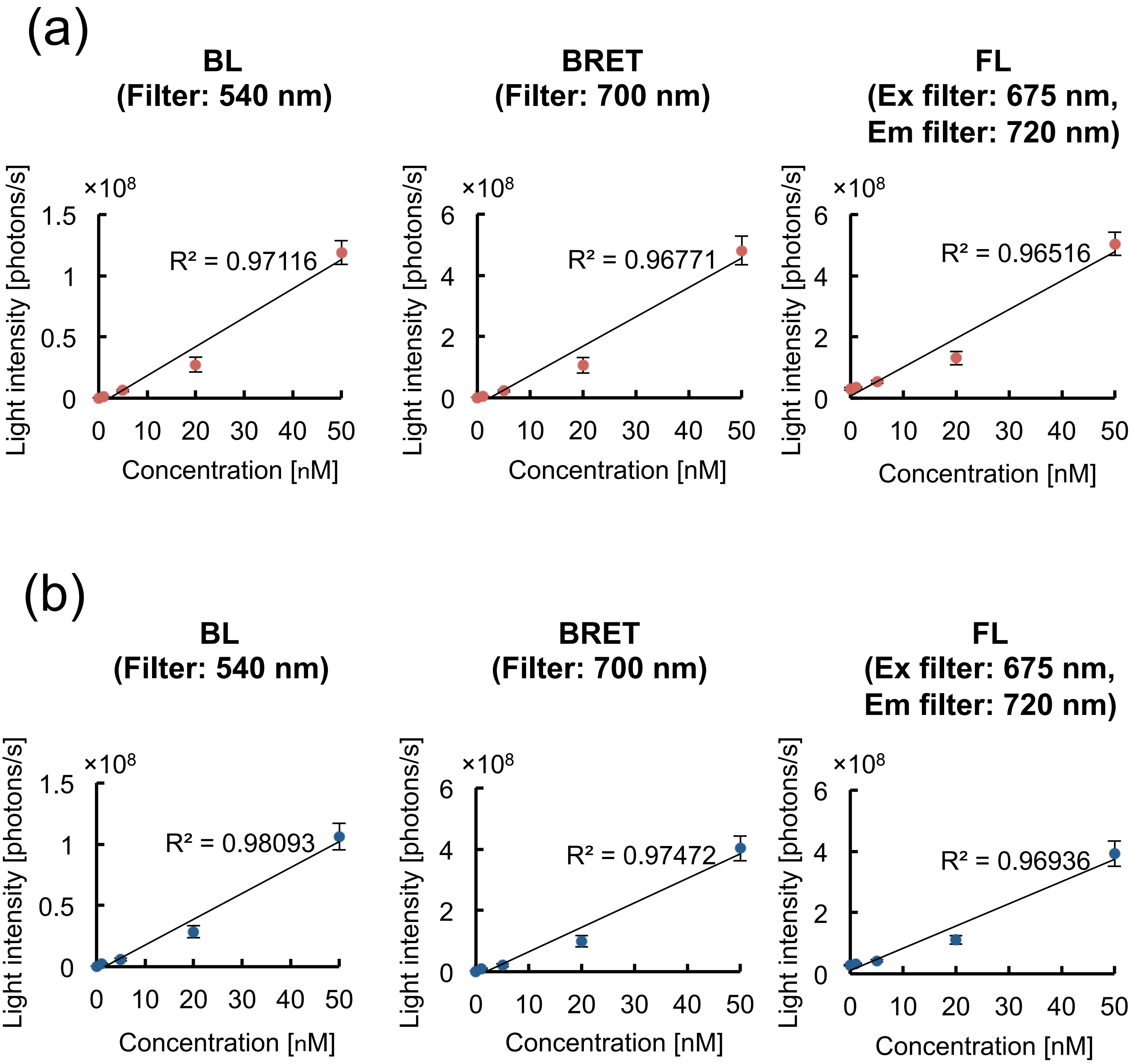

Supplementary Figure S3. Correlations between probe concentration and intensities of light signals. (**a**) POL-AF and CTZ (final concentration of 0-50 nM and 12 μM, respectively) were reacted in 96-well plate and then bioluminescence (BL), BRET and fluorescence (FL) intensity were measured using IVIS-Spectrum. n=3. (**b**) OL-AF and CTZ (final concentration of 0-50 nM and 12 μM, respectively) were reacted in 96-well plate and then BL, BRET and FL intensity were measured using IVIS-Spectrum. n=3.

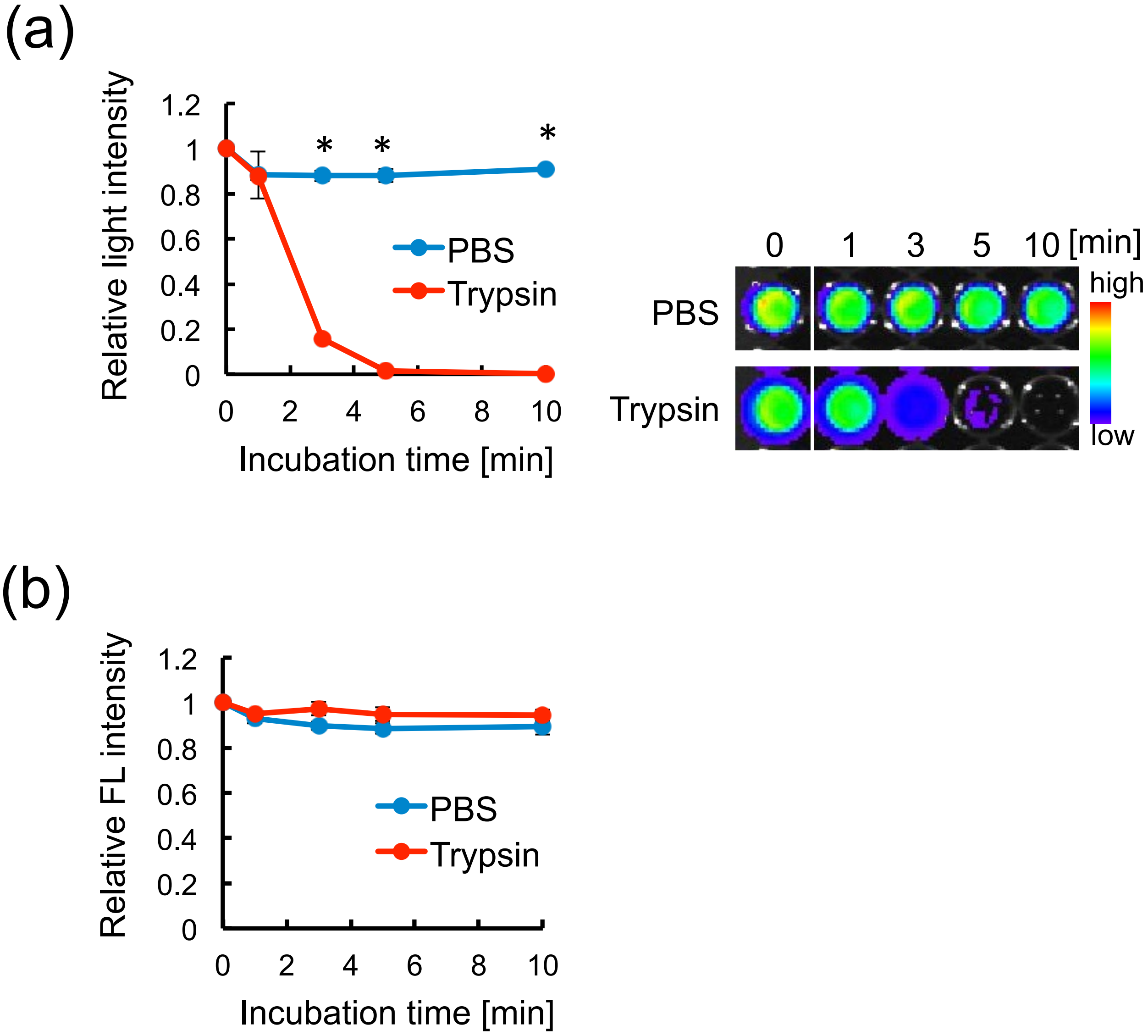

Supplementary Figure S4. Abrogation of BRET in POL-AF by trypsin treatment. (**a**) Trypsinization extinguishes BRET signal of POL-AF. The probe (final concentration of 12 μM) was treated with 1.25% trypsin-EDTA for indicated time and then intensities of BRET signal were measured by using IVIS-Spectrum with a filter of 700 nm. Relative BRET signal intensities to the untreated one (0 h) are shown. n = 3, *$p<0.05$. Representative BRET images of untreated (0 h) and treated probes are shown in the right panels. (**b**) Relative fluorescence (FL) intensities of POL-AF in the same samples as A to the untreated one (0 h) are shown. intensities of FL signal were measured by using IVIS-Spectrum with filters of 675 nm and 720 nm for excitation and emission, respectively.

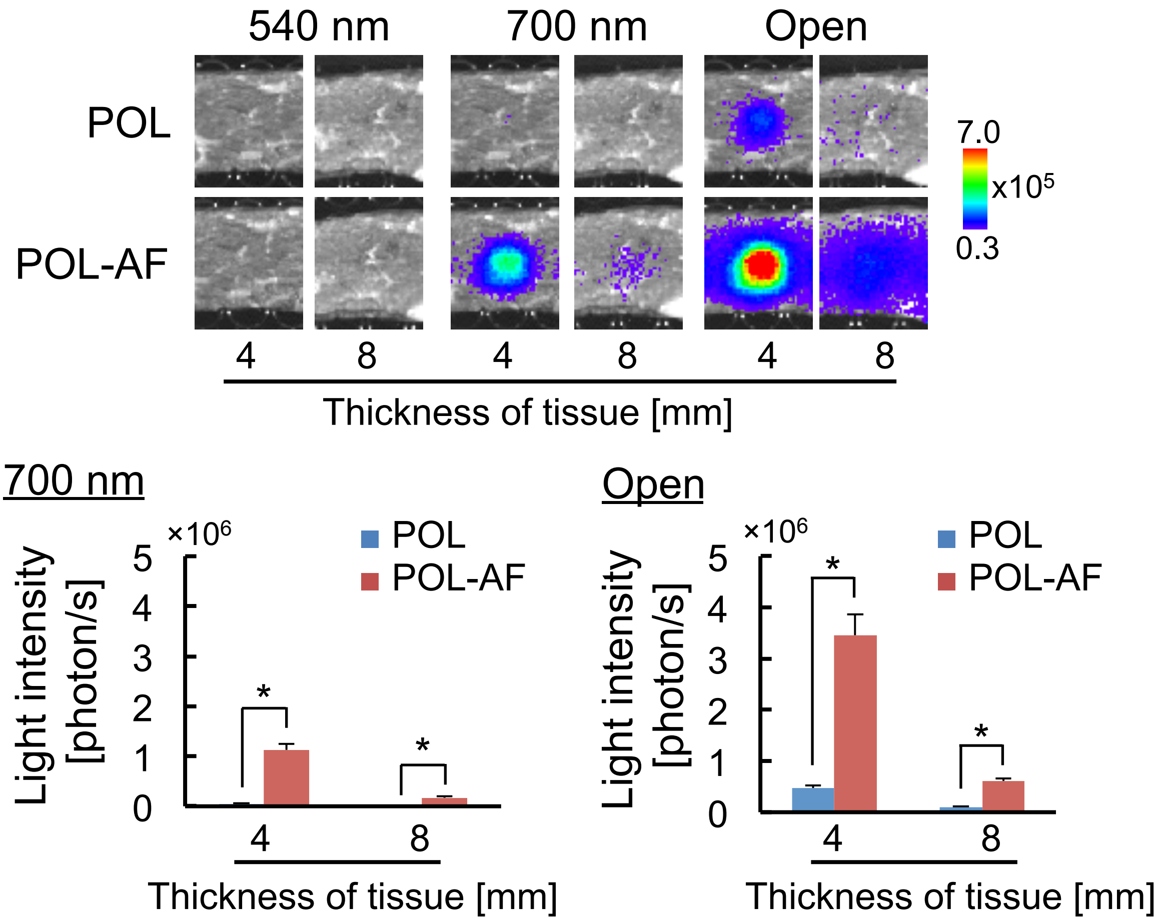

Supplementary Figure S5. Analysis of tissue penetration efficiency of BRET signal. The mixture (100 μL) of POL or POL-AF and CTZ (final concentration of 10 nM and 12 μM, respectively) was in 96-well plate covered with a biological tissue (4-mm or 8-mm thick sliced beef). Representative images of light through the biological tissue (upper) and quantitative analysis of light intensity (bottom) with indicated filters. Light signal was not detectable with the 540 nm filter. n=3, *$p<0.05$. Error bars indicate s.e.m..

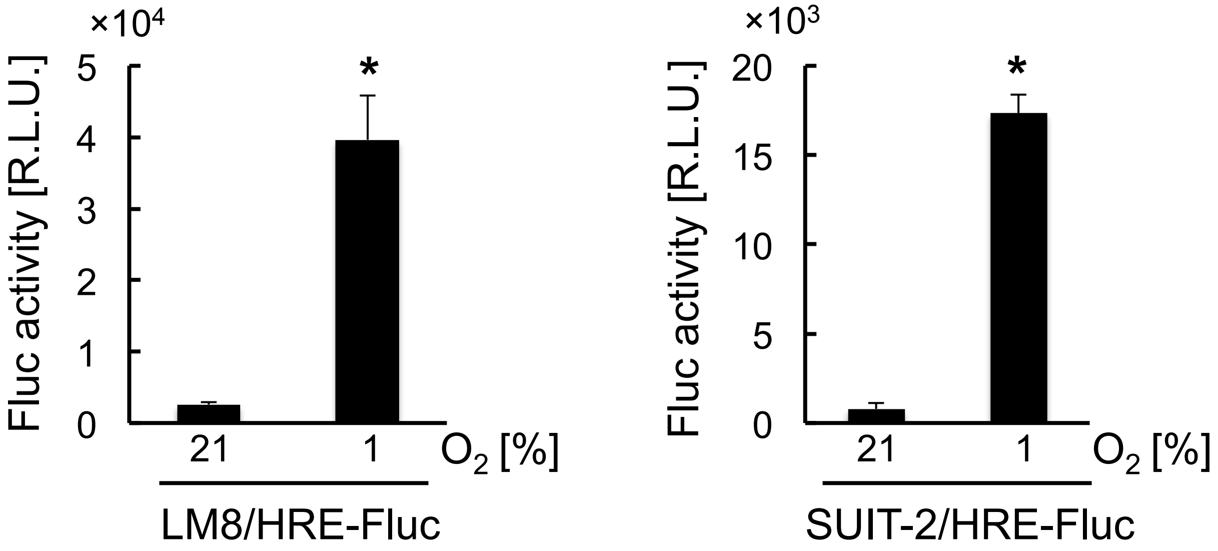

Supplementary Figure S6. Regulation of transcriptional activity of HIF under normoxic or hypoxic conditions. LM8/HRE-Fluc and SUIT-2/HRE-Fluc were cultured under 21% or 1% O_2 for 16 h and then luciferase expression was evaluated by bioluminescence intensity of the cells. n=3, *$p<0.05$. Error bars indicate s.e.m..

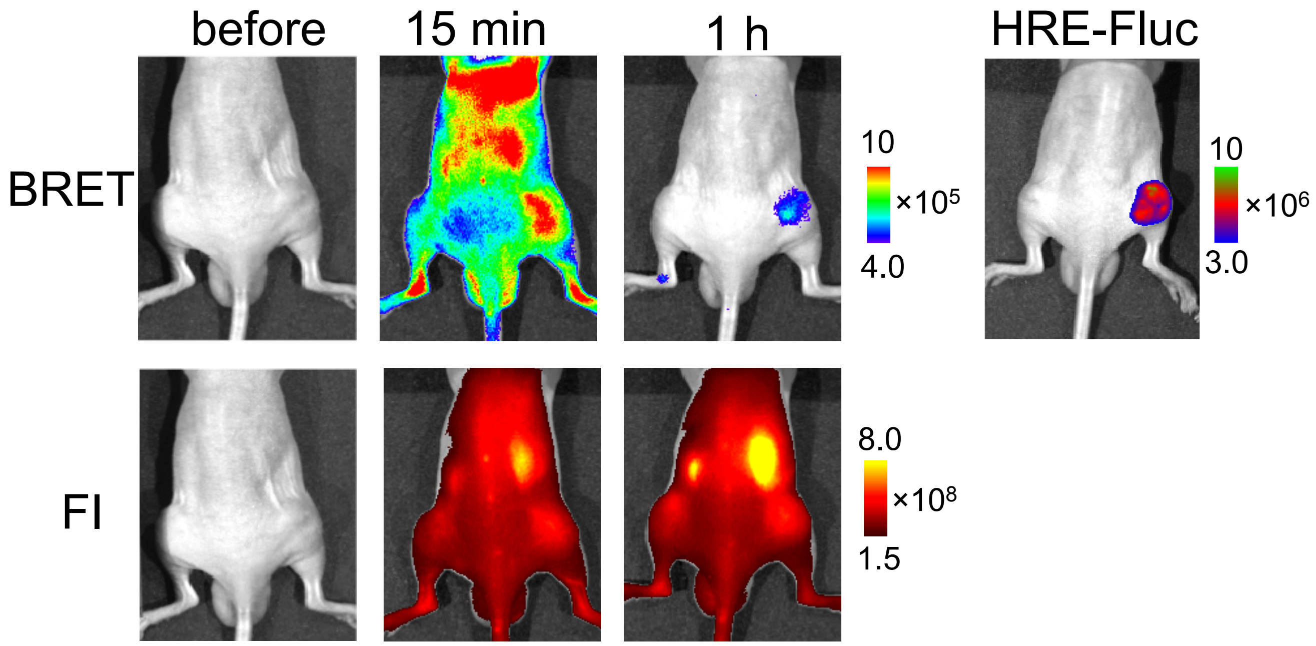

Supplementary Figure S7. Time-course imaging of subcutaneous tumors of LM8. Subcutaneous tumors of LM8/HRE-Fluc were visualized 15 min and 1 h after POL-AF injection.

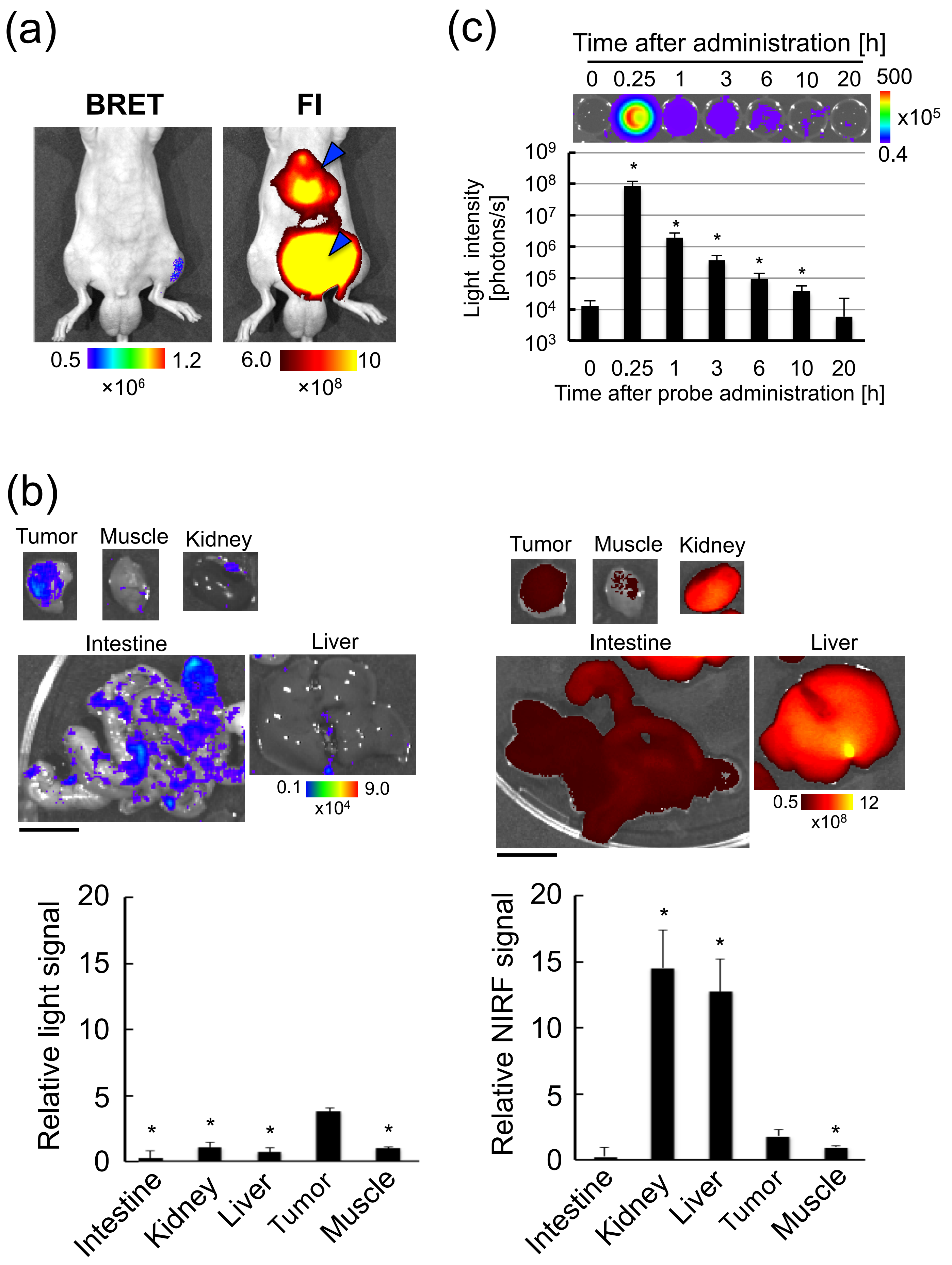

(a)
BRET
FI
0.5
1.2
×10⁶
6.0
10
×10⁸
(c)
Time after administration [h]
0 0.25 1 3 6 10 20
500
x10⁵
0.4
Light intensity [photons/s]
10⁹
10⁸
10⁷
10⁶
10⁵
10⁴
10³
0 0.25 1 3 6 10 20
Time after probe administration [h]
(b)
Tumor
Muscle
Kidney
Intestine
Liver
0.1
9.0
x10⁴
0.5
12
x10⁸
Relative light signal
Relative NIRF signal
20
15
10
5
0
Intestine
Kidney
Liver
Tumor
Muscle

Supplementary Figure S8. Analysis of off-target signals for *in vivo* imaging of POL-AF. (**a**) Representative ventral images of a mouse 1 h after injection of POL-AF. Blue arrowheads indicate fluorescence signals form the liver and bladder. (**b**) Analysis of BRET (left) and fluorescence (right) signals in excretion organs. Mice with subcutaneous tumor of LM8/HRE-Fluc were injected with 10 μg coelenterazine at 1 h after i.v. injection of POL-AF (1 nmol). Relative signals of organs to corresponding muscle signal are indicated in the bottom graphs. Representative *ex vivo* BRET and FI images of major organs are shown in the top panels. n=3, $^*p < 0.05$ to the tumor signals. Bars = 10 mm. (**c**) BRET signal intensity in the blood. Blood sample (5 μL) were obtained from the tail vein at indicated time after i.v. administration of POL-AF (1 nmol). Blood samples in a 96-well plate were mixed with coelenterazine and BRET signals were measured with a filter of 700 nm. n=3, $^*p < 0.05$. Error bars indicate s.e.m..

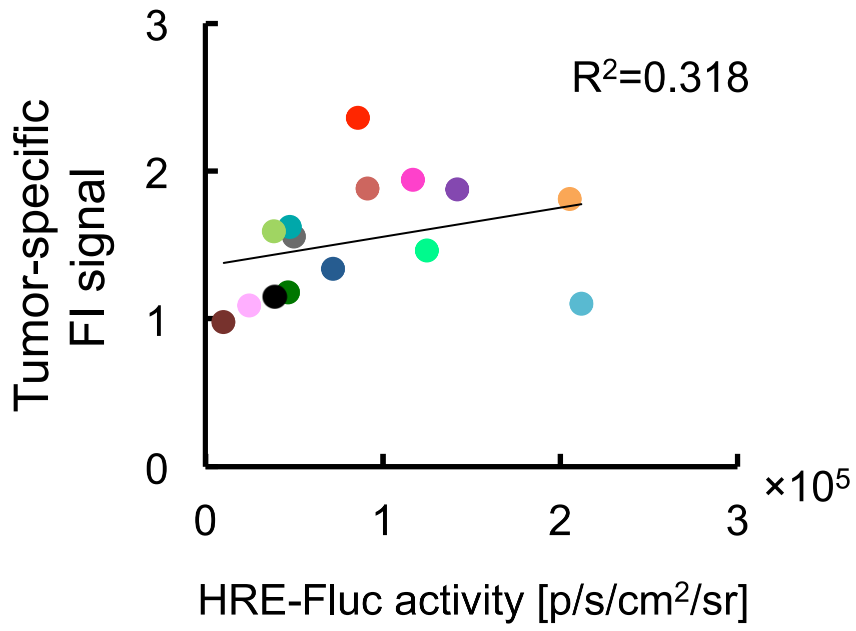

Supplementary Figure S9. Correlation between intratumoral HIF-activity and tumor-specific fluorescence signal of POL-AF. Tumor-specific fluorescent signal (T/M ratio) and bioluminescence from each LM8/HRE-Fluc tumor is plotted by a cycle. The results of tumor-specific BRET signal of POL-AF in the same subcutaneous tumors are indicated by the same colored circles in Fig. 3c. n=17.

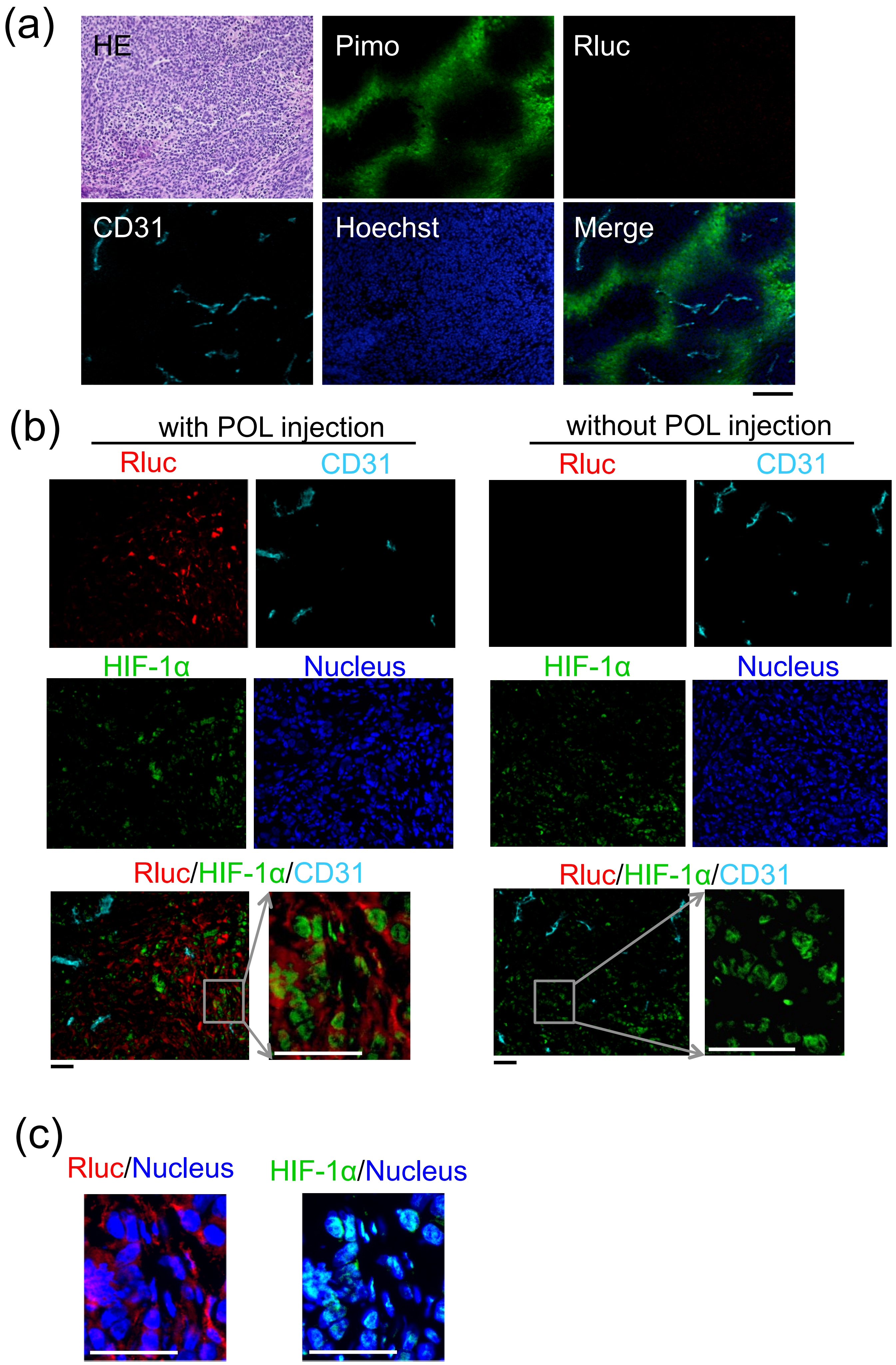
(a)
HE
Pimo
Rluc
CD31
Hoechst
Merge
(b)
with POL injection
without POL injection
Rluc
CD31
HIF-1α
Nucleus
Rluc/HIF-1α/CD31
(c)
Rluc/Nucleus
HIF-1α/Nucleus

Supplementary Figure S10. Immunohistochemical analysis of subcutaneous tumors. (**a**) Immunohistochemical analysis of LM8/HRE-Fluc subcutaneous tumor section without POL probe injection. Serial frozen sections were used for HE staining and immunofluorescence staining. Corresponding data of POL probe injected tumor are shown in Fig. 4. Bar = 100 μm. (**b**) SUIT-2/HRE-Fluc subcutaneous tumor with or without POL probe injection. Sections were prepared from tumors from POL-treated (left panels) or -untreated (right panels) mouse. Tumors were removed 1 h after POL injection. The area surrounded with a squares are enlarged on the right side. Bars = 50 μm. (**c**) Photos of the same enlarged area of POL-treated section as (b) are shown with immunofluorescence staining for POL (Rluc, red), HIF-1α (green) and nucleus (Hoechst, blue). Bars = 50 μm.

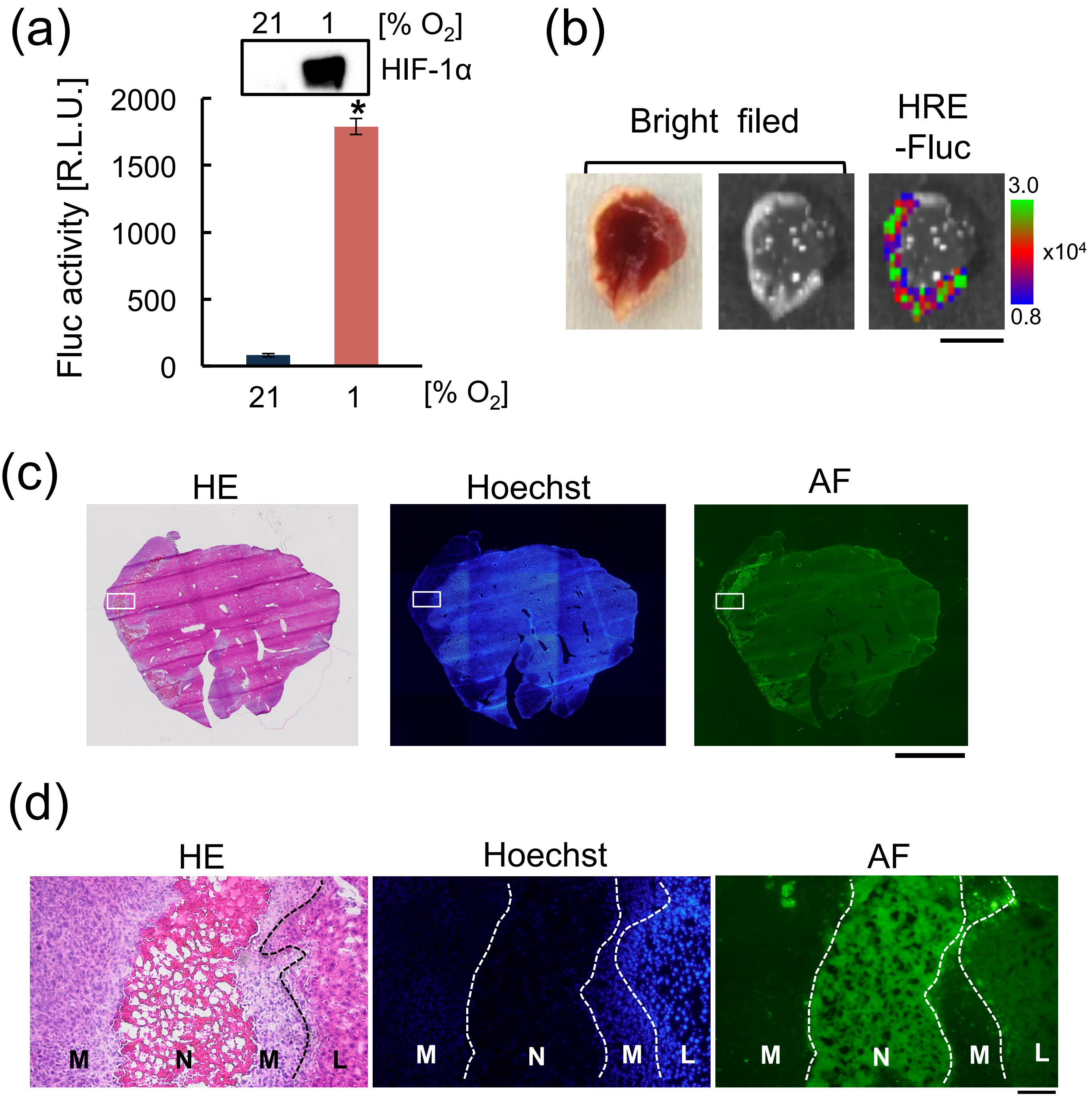

Supplementary Figure S11. HIF-activity in liver metastasis of Colon-26/HRE-Fluc. (**a**) Fluc activity of Colon-26/HRE-Fluc. Colon-26/HRE-Fluc cells were cultured under normoxic (21%O_2) and hypoxic (1%O_2) conditions for 16 h and their luciferase activities and HIF-1α protein expression (top panel) are shown. n=3, * p<0.05. Error bars indicate s.e.m.. (**b**) Typical images of dissected liver metastasis and HRE-Fluc bioluminescence. Bar = 0.5 mm. (**c**) Serial sections of the liver tissue with metastasis shown in (b). Hematoxylin-eosin (HE) staining, fluorescence microscopy of Hoechst perfusion and autofluorescence (AF) in GFP filter are shown. Bar = 1 mm. (**d**) Magnified images of the area indicated by white squares are shown in (c). Limited diffusion of Hoechst dyes was observed in large part of metastatic (M) and necrotic (N) areas compared to normal liver tissue (L). Bar =100 μm.

Supplementary Methods

Stability assay of POL-AF in serum. POL-AF was incubated with 50% fetal calf serum (FCS) at 37 °C and BRET signal intensity was measured at indicated time by using IVIS-Spectrum (PerkinElmer) with a filter of 700 nm.

Biodistribution analysis of POL-AF. POL-AF (1 nmol) was injected intravenously into nude mice harboring subcutaneous tumors of LM8/HRE-luc. The mice were euthanized 1 h after probe injection and major organs were immediately harvested. BRET signal and fluorescence emissions from these organs were measured with the IVIS system using the same parameters for *ex vivo* imaging. For assessment of BRET activity in blood circulation, 5 μL of blood was sampled from a tail vein at indicated time after i.v. injection of POL-AF (1 nmol). Coelenterazine (final concentration of 12 μM) was added to the blood samples and BRET signal was measured with IVIS. Image analysis was performed with Living Image 4.2.

Blood flow analysis in liver metastasis tissue. Colon-26/HRE-Fluc (5×10^5 cells/100 μL) was injected in the spleen of nude mice. Five days later, D-luciferin (50 mg/kg) was intraperitoneally injected to liver metastasis bearing mice 14 min before i.v. injection of Hoechst 33342 dye (1 mg dissolved in 100 μL PBS). One min after Hoechst dye injection, mice were euthanized and liver metastasis tissues were immediately removed. After *ex vivo* bioluminescence imaging of HRE-Fluc in removed tissues, samples were embedded in Tissue-Tek OCT compound (Sakura Finetechnical) in dry ice/methanol baths. Serial cryosections (10 μm thick) were prepared using a cryostat (Leica Microsystems). Serial sections were stained with hematoxylin-eosin for HE staining analysis and directly mounted with Fluoromount™ (DBS) for fluorescence observation.
